# Similia Similibus Curentur: Theory, History, and Status of the Constitutive Principle of Homeopathy *

**DOI:** 10.1055/s-0041-1725061

**Published:** 2021-05-17

**Authors:** Josef M. Schmidt

**Affiliations:** 1 Institute of Ethics, History, and Theory of Medicine, Ludwig Maximilian's University, Munich, Germany

**Keywords:** history of medicine, theory of medicine, homeopathy, Principle of Similars, epistemology

## Abstract

A clear definition of its subject and correct application of its tenets are the basis of any science. Conversely, the want of a unanimous understanding of its constituting principles by the homeopathic community is undermining its scientific practice, research and discussion. To facilitate these, first and foremost the Principle of Similars, *similia similibus curentur* , has to be clarified and assessed in terms of its theoretical meaning, historical development, and epistemological status. Hahnemann's conceptions, explanations, and appraisals were not static but evolved and hardened over the years, especially from 1796 to 1810. While initially he related *similia similibus* to an imitation of similar cures by nature and proposed it as an opposition to *contraria contrariis* , he later generalised it to the treatment of any disease. Whilst originally he considered it to be a hermeneutical principle, or a hint towards a curative remedy, Hahnemann later dogmatised it as the only truth. Considering advances in epistemology and theory of medicine, however, the Principle of Similars may not be assessed as a final truth or natural law to be empirically verified or falsified for good, but rather as a practical maxim, guiding the artist of healing in his/her curing of diseases rationally and individually.

## Introduction

What is homeopathy? This simple question may not only provoke but also remind homeopaths and their critics of the basic structure of exploration by which philosophy and rational thinking began to be scientific. By questioning, e.g. a commander “What is courage”, 1 a priest “What is piety”, 2 or a mathematician “What is knowledge”, 3 etc., it was Socrates who first dared to do what nobody had done before: challenging experts of different disciplines to give an account of the basic conception on which their profession, identity, and purpose of life is based. As a rule, however, none of his contemporaries proved to be able to define their field's subject or leading idea satisfactorily, so that the dialogues either ended in *aporia* or in postponement.

As to homeopathy, what would advocates as well as adversaries of homeopathy say today, 25 centuries after Socrates? Obviously, they ought to have a clear-cut understanding of what they are talking about when they e.g. claim “homeopathy is scientifically proven” or “disproven” or the like and invest huge amounts of resources and vital energy into its practice, research and discussion. Looked at more closely, however, large parts of the homeopathic community still seem to be far from a unanimous conception of what homeopathy is all about, and are rather prone to a pluralism of diverse “homeopathies”—which would of course be the clown of any scientific treatment of the issue. 4

Hence, to shed some medical-historical and -theoretical light on homeopathy's constitution, the following account may provide an outline of: (1) the theoretical meaning of its constitutive principle *Similia similibus curentur* —as conceptualised by its founder; (2) its historical development from Hahnemann's first proposal of a new principle to a kind of final dogmatism—as traceable in his original writings; and (3) a critical assessment of its up-to-date epistemological and medical-theoretical status—as a basis for rational practice, research and discussion.

### The Significance of *Similia Similibus Curentur*

#### The Meaning of the Principle of Similars

According to general (yet mainly superficial) consent, homeopathy rests on some basic constitutive principles, such as drug provings with healthy humans, the administration of single remedies in minimal doses, and the Principle of Similars. The Latin formula for the latter, as used by Samuel Hahnemann (1755–1843), reads *similia similibus* and in its complete form *similia similibus curentur* .

The complete version of this phrase ( *similia similibus curentur* ) can be found in only one passage of Hahnemann's entire literary work (encompassing 27,000 pages) 5 : in the introduction of the Organon (in all six editions, 1810–1842). 6 To be sure, it does not read *similia similibus curantur* , as it is often wrongly referred to in secondary literature, but nowhere found in Hahnemann's writings. Considering the subjunctive *curentur* , as well as the meaning of *curare* (to treat!—not to heal, as it is obvious from the saying *medicus curat, natura sanat* ), it has to be translated as “Likes should be treated by likes” or, understood as an imperative, “Treat likes by likes”.

But what does it mean to treat likes by likes? Especially when considering that “similar” is a term of relation which, standing alone, without context, does not make sense!

#### Similia Similibus Curentur

At the aforementioned passage in the Organon, Hahnemann gives the following explanation for “ *similia similibus curentur* ”: “Choose … in every case of disease a remedy which is capable to excite by itself a similar suffering ( *hómoion páthos* ) to the one that is to be cured”. 7 However, if virtually all diseases should be treated by similars, why does the formula not read *omnes morbi similibus curentur* ?

As Hahnemann further indicated at this point, the new principle was meant to be the contrary of the principle *contraria contrariis curentur* and its therapeutic corollary of palliation. The realm of indication for the contraria principle, however, was *contraria* , i.e. conditions for which a contrary exists, such as warm or cold, dry or wet, according to humoral pathology. So, while cold conditions were used to be treated by warm remedies, and warm conditions by cold remedies, etc., Hahnemann now appeared to suggest the opposite, i.e.: treat warm conditions with warm remedies, and cold conditions with cold remedies. But, strictly speaking, the new formula would then have to be: *contraria similibus curentur* !

Now, if *similibus* means the applied similar remedy, what could the first part of the formula, *similia* , refer to? Because this question cannot be clarified in this passage of the Organon, the other sites with the abbreviated formula must also be considered. Amazingly, the short phrase *similia similibus* also appears strikingly seldom in Hahnemann's entire work: only in three articles of 1796, 1807 and 1808, in four passages altogether (apart from the introduction and preface of the Organon).

#### Proposal of a New Principle

In his article of 1796, “Essay on a New Principle for Ascertaining the Curative Powers of Drugs”, Hahnemann mentioned the Principle of Similars for the first time, in the following context: “Imitate nature, which sometimes cures a chronic disease by another joining one, and employ, in the (eminently chronic) disease to be cured, that medicine which is able to elicit another artificial disease as similar as possible, and the former will be cured: *similia similibus* ”. 8 9 Thus, *similia* would mean that the disease to be treated should be similar or analogous to those diseases which nature at times heals by adding another (similar) one.

In his article of 1807, “Pointers to the Homeopathic Use of Drugs in the Former Practice”, Hahnemann again contrasted treatment by similars with palliation and treatment by contraria. Here, however, he insinuated that allopaths would—by the principle of contraries—not just treat those conditions that have a contrary, i.e. *contraria* , but any. 10

In his open letter to Hufeland in 1808, Hahnemann reinforced that allopaths were just treating *contraria* in a palliative way, while at the same time he himself enlarged the indication for the “curative administration of remedies ( *similia similibus* )” to all “eminently” “prolonged diseases”. 11 12 Thus, it seems that the first part of the formula, *similia* , had lost its sense. In fact, henceforth both principles were used by Hahnemann in the sense of *omnes morbi similibus curentur* versus *omnes morbi contrariis curentur* .

This overworking of the maxim *similia similibus* on the part of Hahnemann beyond its conceptual scope may be the reason he had invoked it so seldomly in his published works. Also in the introduction and preface of the Organon the short phrase *similia similibus* occurs only in a few passages (never in the main part of the Organon and never in all six editions). 13

### Hahnemann's Development and Dogmatisation of the Principle of Similars

#### Historical Background and Hahnemann's Attitude

To comprehend how Hahnemann came to his reformulation and amplification of meaning of the Principle of Similars, a short look at the background of Hahnemann's work may be helpful. It may be revealing to trace Hahnemann's change of attitude within some 10 years in terms of how he presented his new principle: from a modest first proposal of a hermeneutic principle (1796), to a progressive extension of its range of indication (1801–1805), to a conclusive certainty of having found the only true and possible way of healing (1807).

In the course of profound political, social and economic change, such as the French Revolution, emancipation of the bourgeoisie, and early industrialisation, as well as intellectual movements, such as Enlightenment, German Idealism and German Romantic, by the end of the 18th century, representatives of all sciences attempted to expand the realm of a modern rationality in their disciplines as far as was possible. Just as Immanuel Kant (1724–1804) tried to raise philosophy to the rank of a rational science, physicians endeavored to do the same for medicine.

What Hahnemann, as a protagonist of that era, had achieved in this respect is remarkable and hard to better in terms of rationality and relevance for practice. This is not least because Hahnemann went to work with an ethical severity and a noble concept of mankind inspiring his critique of and attempt to reform medicine. The physician's ethos and task to cure sick humans prohibited Hahnemann both to conduct medicine according to primarily economic considerations as well as to let pass unchallenged the sloppiness of physicians and pharmacists, who administered mixtures of widely unknown substances for abstract names of diseases. Nor was it compatible with the dignity of medicine to rely on superstition, chance findings, crude empiricism, speculations of natural philosophy, romantic infatuation, or mechanic–materialistic reductionism. In distinction from all competing approaches and in permanent reference to practice, Hahnemann instead suggested plain pragmatic measures, such as the usage of single remedies, drug provings with healthy humans, differentiation of disease states and effects of drugs in terms of symptoms, and self-manufacturing of drugs. 14 15

Only by these measures, and thereby created transparency, was it possible for Hahnemann to discover “things which otherwise [he] never would have seen”, 16 17 such as the systemic effect of minimal doses of remedies, for which since 1797 he used the term “dynamic”, 18 19 20 21 22 23 or the similarity of certain drug symptoms with certain disease symptoms.

#### A New Hermeneutic Principle

From initial observations of a correlation between drug proving symptoms and cured disease symptoms, the idea arose of a practically applicable regularity. In 1796, Hahnemann felt entitled to propose a new “principle” or “key”, according to which “gradually for each, more especially for each chronic disease, an appropriate specific remedy may be found”. 8 9

In 1801, Hahnemann described how, in 1799, he had also applied his “new synthetic principle” to the treatment and prevention of an acute disease, scarlet fever. 24 25 Still he considered his “new principle” to be simply “a hint to learn to look at diseases from a perspective that points to the appropriate remedy, nearly unambiguously—a hint to find, according to the positive nature of the remedies, the diseases for which they must be suitable”. 26 27

In the same year, Hahnemann declared that—apart from so-called stable diseases that have an obvious cause or arise from an infection with a miasm, such as syphilis or scabies—all other diseases differ from each other so manifoldly that each of them must be seen as an individual. Although they lack a direct causal therapy, there is a “shorter more natural way” to find a remedy for these conditions. 28 The application field for the Principle of Similars, which had been insinuated thereby, seemed to have been extended here to all diseases with unknown causes.

#### The Principle as a Factual Claim

In 1803, in a monograph on the effects of coffee, Hahnemann for the first time framed his heuristic principle in the form of a factual claim, when he wrote: “When the medicine is employed in cases of disease that have an almost congruent similarity with the alterations that the medicine is capable of itself producing (in the healthy body), a thorough cure will take place”. 29 30

In 1805, in a treatise to the broad public, Hahnemann declared as a *desideratum* of the epoch a “certain, unfailing” medicine which has to “thrive towards a science” that can be “learned and taught”. At the same time he emphasised that—regarding the treatment of chronic diseases—natural sciences, especially chemistry, may not be of great help, since “what is salutary or hurtful” in medicines is “out of its sphere of vision”; and chemistry is “overmastered by vitality” in the human body. 31 32

In 1805, his book “Medicine of Experience”, the forerunner of the Organon, claimed to offer a solution for the difficulties of medicine at that time. To substantiate why his new doctrine may be appropriate for all the disparate individual diseases lacking a stable cause and name, Hahnemann now introduced several theoretical postulates into his hitherto mainly empirical approach. For example, “the inner nature of every disease” was considered to express itself in the “available signs”—meaning that with the “perceivable signs” “the disease itself is found”. “For the sake of healing” nothing else would be “necessary to be known” by the physician, a circumstance thought to be guaranteed by the “wise and kind Creator” who would “not leave his children helpless or ask more of them than they can achieve”. Another tenet—that cures of diseases can only occur by curative, never by palliative remedies—was explained by a theory according to which in the human body two stimuli cannot “exist beside each other and at the same time”. If two stimuli are dissimilar, they would suspend each other. If they are similar, the weaker one would be removed or annihilated by the stronger one. 33 34

#### Generalisation and Loopholes

Under these theoretical premises, Hahnemann now generalised his initially hermeneutic principle to factual theses and, henceforth, recognised no borders regarding the scope of its application. The range of indication of curative remedies was therefore explicitly extended to also include acute diseases.

To underline his claim of validity and exclusiveness of healings by curative remedies (according to the Principle of Similars) scientifically, Hahnemann began at that time to quote evidence from literature or from daily life. The examples ranged from a citation of Hippocrates to the treatment of overheating by a “sip of brandy”. 35

Some loopholes, however, were left in this otherwise closed system. For instance, the symptoms of a remedy may not only be found in provings with healthy humans, but “even in diseases”—however, this would be “a topic of higher contemplation, reserved only to masters in the art of observation”. Hahnemann bluntly admitted that his first materia medica ( *Fragmenta de viribus medicamentorum positivis* ) had come about in this (mixed) way. And still, not only the picture of the disease in its signs, but also the knowledge of its occasion and original cause, was considered to pertain to the rationale of healing.

#### Only Truth and Natural Law

Whilst in 1805 homeopathy had been founded as a distinct doctrine or system of medicine, it was only in 1807 that Hahnemann coined its proprietary name, when he defined: “Homeopathic is, what has the tendency to generate a *hómoion páthos* , a similar suffering”. Contrary to 1796, Hahnemann's initially hermeneutic principle *similia similibus* had now advanced to be considered the sole “truth”, the “only way to heal diseases gently, quickly, and lastingly”, or the “most rational and perfect of all ways of healing”. As evidence, Hahnemann quoted more than 400 authors confirming his claims by taking the example of almost 50 medicines. 10

Also in his open letter to Hufeland of 1808, Hahnemann spoke of the “only salutary way”, of the “only way which leads to health with certainty”, of “truth” and—for the first time—of a “natural law”. He now deemed “the proposition irrevocable” that “any disease” can be “cured” homeopathically, solely based on its “coincidences and sensations”. 11 12

#### Rational Therapeutics

In the “Organon of the Rational Doctrine of Healing” of 1810, Hahnemann, for the first time, called his new method by the noun “homeopathy”, and claimed that it is based on the “homeopathic healing law”, the “homeopathic natural law”, and the “eternal invariable law of homeopathy”. 36 With these notions the dogmatisation and absolutisation of the new doctrine had reached a peak that could not be excelled in later writings. Only rhetorical enhancements may at best be found, e.g. in 1819, when Hahnemann claimed that the homeopathic “healing business will come close to the mathematical sciences in terms of certainty”, 37 or in 1833, when he declared that homeopathy is the “only right”, “only possible”, and “true, best” way of healing—as certain as “between two given points only one straight line is possible”. 38

Not even the challenge by therapy-resistant chronic diseases could unsettle Hahnemann in his conviction that “the doctrine itself rests on irrefutable pillars of truth and … will be so forever”. 39 40 With his theory of chronic miasms, published in 1828, he rather expanded the range of application of the Principle of Similars, from a phenomenological treatment of complex disease states whose causes were not discernible, to the claim of a (dynamic) therapy even of causes, such as the chronic miasms psora, sycosis and syphilis. 41

### The Epistemological Status of Hahnemann's Principle of Similars

#### Controversial Reception

Hahnemann's claim of having found the only true and possible way of curing all diseases (except for surgical and life-threatening emergency situations) did not remain unopposed. From the 1820s, it was in particular the question of the scope of application of the Principle of Similars that caused first schisms and emancipation movements within the young homeopathic community. As it may be shown in the following, most of the ensuing controversy was conditioned by the parties' misunderstanding regarding the epistemological status of their claims.

Generally, the frame of thought of that time was predestined by the alternatives induction versus deduction (or empiricism vs. rationalism), and by a lack of subsequent categories for coping with anomalies. Hence, failure could only be ignored, suppressed, rationalised or warded off in certain ways, for example by rhetoric, polemics, immunisation, reproach of heresy, imputation of “adulteration” through “allopathy”, etc. Thus, a sober discussion seemed impossible. 42 Indeed, Hahnemann consistently continued his assertion of having come to his “maxims of experience” ( *Erfahrungssätze* ) 43 44 and “doctrines” ( *Lehrsätze* ) 45 46 merely “by observation, reflection and experience” ( *Beobachten, Nachdenken und Erfahrung* ). 47 48 Having proved successful, he argued, they must represent the “truth” or “natural laws”.

This stalemate and disability of communication and structural intolerance towards competing methods, in which homeopathy seems forever to be stuck, may meanwhile be resolved by taking into account new approaches of epistemology and theory of medicine.

#### Theory of Science

After neither the positivism of the Vienna Circle around Moritz Schlick (1882–1936) 49 nor the critical rationalism of Karl Popper (1902–1994) 50 51 could survive, the liberation of epistemology from any kind of methodological bondage by Paul Feyerabend (1924–1994) 52 and the criticism of ideology of knowledge for the sake of control by the Frankfurt School 53 54 led to the consensus that science may not generate anything like absolute knowledge, but rather be comprehended as a social process. 55

Kurt Gödel (1906–1978) in 1930 had mathematically proven the theorem of interminability of any axiomatic system, 56 Ludwig Fleck (1896–1961) had, in 1935, pointed out the importance of the tradition of apprenticeship and perception for the “style of thought” of any “thought collective”, 57 58 and Robin Collingwood (1889–1943) had shown in 1940 that any science contains “absolute presuppositions” which are not deducible from itself. 59 Thomas Kuhn (1922–1996) in 1962 had explained that the process of science is running linearly and rationally only within certain plateau phases, in which scientists orient themselves by commonly accepted paradigms; however, intermediately, it also acts disruptively or revolutionarily, resulting in radical redrafts with a debasement of all former achievements. 60

From the perspective of this rather deconstructive side of modern epistemology, Hahnemann's claim to absoluteness (since 1805) appears—systematically—as untenable presumptuousness and—historically—as a typical brainchild of his time, in which the paradigm (or style of thought) prevailed to preferably explain everything from one principle, to set it up as “the truth”, and defend it against competing claims of truth.

#### Abduction as a Creative Act

To also positively appreciate Hahnemann's achievements and to elucidate the status of their concept in a clearer manner than he and his contemporaries were able to, it may be necessary to resort to Charles Sanders Peirce (1839–1914) and his concept of “abduction”, which he developed over a century ago, 61 drawing on Aristotle's notion of “apagogé”. 62 Contrary to induction, which merely arrives at probability but never at final physical laws, and contrary to deduction, which is logically stringent and irrefutable but does not yield new insights, abduction consists of a creative act leading from the observation of relevant details to the awakening in one's mind of an idea which connects them in a sensible way. Because a new theory, however, can in no way be present in encountered data, its discovery (by abduction) cannot be systematically reconstructed, algorithmised, operationalised or simulated. That is why this notion and this process—contrary to induction and deduction—is strongly neglected in the conventional theory of science. 63

Although there is no rule according to which abduction, i.e. the finding of a satisfying structure for the explanation or connection of single points of data, may be learned, in the scientific process as well as in medical practice it plays an elementary role. For example, to bring down disparate symptoms of a patient to a common denominator, i.e. under a diagnosis, the physician often needs an inspirational idea, just as the researcher to whom—like Isaac Newton (1642–1726) before his legendary “apple”—an idea suddenly manifests itself to comprehend numerous incoherent observations under one new consistent viewpoint.

What Hahnemann accomplished when he established the Principle of Similars (in his version) as the “key” or “new principle” to align—within the state of knowledge of the medicine of his time—the treatment of diseases to a hitherto unprecedented guiding principle, may neither be sufficiently grasped by induction nor by deduction, but rather by an act of ingenious abduction. 64 65

According to the recent understanding in the theory of science, abductive conclusions always need to be checked recursively, by deducing further particular cases. Because of the error-proneness of abduction as a form of conclusion, from any theory achieved in this way, concrete expectations are to be formulated (by way of deduction), whose accordance with particular observations has again to be proven by the way of induction. Thus, ideally a circle is formed that keeps the scientific process running. 66 At no point may it be justified—and this is the difference to the mentality of Hahnemann's era—to terminate the process and speak of “eternal truth” or the like. Nevertheless, among scientists there may be personalities who are inclined towards one of the three (complementary) forms of conclusion to a special degree.

#### Hahnemann as an Artist of Healing

Hahnemann's strength may well have lain in his creativity and disposition as an artist, hence in the realm of abduction. When he was still a pupil and student, he was exempted by his teachers from regular requirements and was permitted to teach himself the subject matter autodidactically and however he preferred. As a young physician he made several chemical and medical discoveries, and during his time in Leipzig, when he renamed the second edition of the Organon to “Organon of the Art of Healing” ( *Heilkunst* ), he called his wife Henriette the “noble companion of his life as an artist” ( *Künstlerleben* ). 67

Of course, Hahnemann did contribute significantly to the areas of induction and deduction as well, especially in his most rationalistic phase (1805–1810), when he tried to translate his ideas into the notions and ways of thinking of his contemporaries, and establish them theoretically as “rational therapeutics”. Remarkable as his semiotic, stimulation-based, and teleological theories and explanations may have been in the context of the scientific discourse at that time, for homeopathy as a practice having worldwide therapeutic relevance even today, they may prove to be secondary or even expendable. What remained, what has spread, and what is still yielding fruit, may have been passed on—scientifically largely unknown until the present day—less by his theories but rather by his practical instructions.

A closer look at the *materia medica homoeopathica* may for example show that even Hahnemann himself—contrary to his rational public image—did not only use pure proving symptoms of drugs, but also so-called curative effects and observations of patients: a practice that—strictly speaking—had relativised his own theory. 68 Hahnemann's theory of primary and secondary effects was partly adopted, partly rejected, by later homeopaths—without having a crucial impact on practice. 69 Also the term “miasm”, used by Hahnemann in a specific sense, today “within homeopathy, is applied in so many different meanings that it might be cancelled from the homeopathic vocabulary without significant damage in communication” (according to a former president of the German Homeopathic Association). 70 Apparently, successful homeopathic practice may be widely independent from the theory taught by Hahnemann and his successors.

#### Hering as a Sceptic of Theories and Artist of Healing

Constantine Hering (1800–1880), the patriarch of homeopathy in the United States and a strict Hahnemannian throughout his life, in 1836, in his preface to the first American translation of the Organon, confessed that he “never accepted any theory in the Organon in the way it was given there”, 71 and declared in 1861, that to understand Hahnemann, one has to “consider his direction as that of an artist” and “recognise him as a very powerful artistical spirit”. 72

Regarding the relationship between art and science, he said: “In history science always comes later than art, it yields nothing whole, perfected in itself, but rather cuts asunder with its anatomical knives… All this is in order; the artist, who has another task, however, should be judged as such”. Far away from ignorant hostility to science, Hering still referred to its limits: “Although the striving for certainty [and] for science is always laudable, nay unrefusable, it must not destroy the art”. 72

When Hering stood up for the art of proving, observing, taking drug pictures, and healing, this position proves compatible with recent developments in the theory of medicine, which, since several decades ago no longer considers the status of medicine—as it was in the preceding 150 years—as an applied science, but rather as a practical science *sui generis* (in its own right). On the grounds of the structural difference between knowledge and action, purposeless science, which is about pure knowledge, may never be the basis of a reflected self-conception of medicine which has a clear purpose: to help sick human beings. Its scientificity may therefore only consist of developing criteria for purposeful actions that are practically viable. 73 74

#### Similibus and Contrariis

Before medicine, in the 19th century, had set about to see itself as an applied science (in misjudgement of its genuine practical nature), Hahnemann had already perfected the programme of medical theory prevailing today. As a true artist of healing, in his aspiration for perfection of the art of healing, by means of his fortunate abduction, he set about to discover a way to cure diseases quickly, gently, and permanently, and to give concrete instructions for this.

To what extent this way of healing may be comprehensible “with clearly understandable reasons”, whether it is teachable and learnable, whether it is the only possible, the shortest, most reliable and least adverse way: these and other questions could arise only subsequently to the primary creative act of opening this new area of research. In fact, they are less concerned with the practical, goal-oriented artist, than with the rationalist, who is tempted or forced to prevail—within the contemporary scientific discourse—against refutations by advocates of competing systems, on the level of rational notions and binary logic.

The difficulty in which Hahnemann found himself with his balancing act between propulsive experimental practice and rationalising theory lagging behind, finds its expression sharply in another quote by Hering: “ *Contraria contrariis* is in theory the only right, but lacks in practice the good works. *Similia similibus* is practically and artificially the best rule, but in theory is no good and lacks all scientific determination”. 72 Long before opponents noticed this, Hering claimed that he had written in 1834, *nota bene* as a strict Hahnemannian: “Both permeated as one, make a human, like the right and left side together; through this then, but only then, all possible may be achieved”. 75

#### Hahnemann's Hypotheses and Postulates

Such a clarified, sovereign distance towards theories that Hering was displaying from America could, in fact, not be expected from Hahnemann, not least because of the tenseness of the German culture of conflict. Yet, Hering always maintained that Hahnemann's “first keen deed” cannot be overestimated.

For Hahnemann, until the end, it was seemingly obvious that his cures (as well as the cures of his disciples who followed his instructions) were based on his theory, according to which cured patients had symptoms that were similar to those that the remedy had elicited in drug provings with healthy humans. To theoretically underpin the Principle of Similars conceived in this way, Hahnemann, from the beginning, used auxiliary hypotheses, such as the concept of primary and secondary effects, of palliative and curative action, and from 1805 postulates, such as the unity of the organism in which two stimuli cannot coexist at one time, the identity of the inner essence of the disease with the totality of symptoms, the unnecessity to know the causes of the disease (because of the wisdom and kindness of the Creator), the invariable superiority of strength of any drug effect over any stimulus of disease, and from 1829—in the context of his theory of chronic miasms—even the imputation that the healing attempts of the vital force would, without assistance by homeopathy, achieve nothing more than “a kind of allopathy”. 76 77

These and other theoretical explanations by Hahnemann—since they were not recognised and resolved as such—ensured fiery academic controversies and ultimately a 200-year-literary tradition of criticism and apologies.

#### Medicine's Practical Task

As suggested by recent developments in the theory of medicine, medicine in general and homeopathy in particular may be well advised, regarding their self-conception, to think of themselves not in terms of contingent theories, but of their practical task and the concrete tools at their disposal. For homeopaths, these consist of the directives of Hahnemann and his successors regarding a detailed individualising case-taking with emphasis on subjective changes of affectivity, a differentiating study of drug effects according to localisation, sensation, modalities and concomitants, and a hierarchisation of peculiar and characteristic signs and symptoms.

This has been—from a pragmatic perspective—the common denominator of homeopathy for the past 200 years. To have found, developed, and defended these practical guidelines may well be assessed as the work of an “empirical genius”, as Hahnemann had once been labeled by Rudolf Flury (1903–1977). 78 On this, and only on this, rests the therapeutic success attainable in medical practice.

Whilst the healing artist's primary interest is to cure sick humans by obeying practically approved rules, the scientist, however, may be anxious to determine the possibilities and limits of the respective procedure. In turn, by means of theorising and specifying the range of indication, this may also prove advantageous for the artist of healing and his/her patients. But since the scientist may experience the possibilities and limits of a method of healing only then, when he/she becomes an artist of healing himself/herself, i.e. masters the art to perfection, such an undertaking equally hits a structural and categorical wall.

#### Hippocrates on the Principle of Similars

Historically, one of the first reflections of this *aporia* of medical theory is recorded from the circle around Hippocrates (460–370 BC), the ancestor of occidental scientific medicine. In the Hippocratic writing “On the Places of Man” ( *Perì tópon tón kát' ánthropon* , *De locis in homine* ) three different principles of treatment were being examined regarding their generalisability: the Principle of Similars, the Principle of Contraria, and the principle, “sometimes this, sometimes that”. 79

Contrary to Hahnemann who quoted this book—albeit selectively—in his “Medicine of Experience” 80 81 and in the introduction of the “Organon” (in all editions), 82 83 the Hippocratic author came to the conclusion that in medicine there cannot be any hard rule that would apply always without exception. Everything rather depends on the context, or on the unique moment, in Greek “ *kairós* ”. 84 Hence, principles and theories may only have an instrumental character, and their workmanlike selection and application to the individual patient is up to the judgement of the artist of healing. 85

#### Hippocrates and Hahnemann

The artist of healing—and thus Hahnemann—would have the right to abide by certain principles, but at the same time would also have the duty to disregard them—as far as the individual circumstances, that are irreducible to a simple scheme, would require it.

The recognition of the latter aspect, the complexity and incomputability of “our organism which is intimately connected with and in conflict with all parts of the universe”, 86 87 had also been the starting point of Hahnemann's practice. Only thus he could come to the assessment that under these conditions neither speculative classification of diseases nor crude empiricism nor natural scientific reductionism could help. What is therapeutically relevant, he concluded, may only be the signs and symptoms to which both expressions of diseases as well as effects of drugs are related, and thus may be matched to each other. 88

Hippocrates would have reserved the right to apply the Principle of Similars—as a hermeneutic rule for appropriate treatments—on some occasions, and not on others, according to the individual case. Hahnemann, however, as a child of a time of rationalism and the romantic quest for the absolute, had become set on the claim to have found the only true way of healing by his re-formulation of the Principle of Similars.

#### Lessons from the Theory of Medicine

After a 200-year-discursive fixation of medicine on cognitive and theoretical aspects of medical science, the recent development in the theory of medicine may again offer a chance to emphasise and support the constitutive practical nature of any kind of healing art. Applied to homeopathy, this would mean to categorically distinguish on the one hand the practical instructions and maxims for the treatment of sick humans, which were found by Hahnemann by abduction and may be recursively scrutinised, i.e. confirmed, modified or discarded—in practice—anew with every patient; and on the other hand the theoretical models which Hahnemann believed he had to establish in his day to approximate medicine to the ideal of an *a priori* certain cognitive science, an endeavor which was then deemed to be progressive. As a child of his time and under the spell of overheated controversies, Hahnemann was not beyond mixing or confounding these two spheres.

From the perspective of theory of medicine, Hahnemann's merit for medicine may consist much more in the practical foundation of a salutary method of healing than in his intended re-definition and re-combination of general theoretical principles and theories. Just as the lived and experienced practice of Christianity may rest on a few irrefutable basic maxims (commandment of love, mercy, forgiveness, etc.) that will retain their validity independent of the outcome of theological controversies on tenets and dogmas introduced by councils of the clergy (consubstantiality vs. homoiousios, eucharistic controversy, immaculate conception, etc.)—analogously also the lived and experienced practice of homeopathy should not be naively equated with Hahnemann's theoretical models (artificial disease, biphasic effect of drugs, identity of symptoms and disease, etc.). Instead, both spheres should be analysed and appreciated separately and understood in their context and interaction.

## Conclusion and Outlook

Considering that, from the perspective of theoretical and historical reconstructions, Hahnemann's conceptual elaboration of his version of the Principle of Similars proves to be shifting and dependent on changing contemporary settings, merely philological and cognitive approaches to an understanding of this constitutive principle of homeopathy may not suffice to grasp its epistemological status and practical significance. Though innovative and progressive, Hahnemann's thoughts were clearly driven by, and achieved within, the frame of presuppositions and paradigms of his time, such as the ideal of medicine as a mathematical science based on natural laws, consistent theories, and irrefutable truths. They were thus caught in the trap of the then prevailing style of thinking of the absolute.

From an up-to-date epistemological perspective, however, rather than being a quest for plain truth, cognitive sciences may today be seen as social processes, encompassing perpetual cycles of abduction, deduction and induction, and as such being averse or immune to closure. Nevertheless, what seems to be constant and true across the centuries and millennia since Hippocrates is the classical insight into the nature of medicine as a practical science *sui generis* or healing art, whose principles and theories may only be instrumental in respect to the primacy of its beneficent goal, to be applied or modified according to the need of the individual case and situation. 89

Therefore, homeopaths (as well as their critics) may be well advised to refrain from dogmatism and sophism, and rather adopt a critical distance towards, and a pragmatic dealing with, principles and theories. Eventually, Hahnemann's initial proposal of a new hermeneutic principle would seem to come closer to a solid, valid and durable conception than his later attempt at connecting to a specific scientific discourse.

All in all, 200 years of quarrel about Hahnemann's ambitious theoretical claims may nonetheless have had an important historical function. As an irony of history, it may have been crucial that—even though neither Hahnemann's theory nor his claim to absoluteness may be tenable in terms of modern theory of science—it was by these contentions that homeopathy has ever since polarised and inflamed minds and spirits. And, paradoxically, thereby it may have everlastingly ensured—as if through trick of reason 90 91 —the passing on and proliferation of its ostensibly plain, but practically highly unique, essence. It may have guaranteed—mediated through an uninterrupted chain of polemic, critique and apologetics—that also the comparatively unspectacular gist of homeopathy, i.e. its most helpful methodical directions with regard to practice, were passed on safely from generation to generation over all continents and cultures.

Ultimately, the strength of the Principle of Similars indeed may not lie in a supposed consistency of infallible deductions or inductions and their logical proof or justification or the like, but rather in being an ingenious therapeutic tool, found and established by a brave and efficient abduction. It may best be understood as an invaluable practical maxim guiding the artist of healing in his or her curing of disease rationally and individually. To be sure, without it, myriads of cures worldwide may never have been accomplished.
